# Antimicrobial susceptibility of *Neisseria gonorrhoeae* isolates in Vientiane, Lao PDR

**DOI:** 10.1016/j.jgar.2017.12.001

**Published:** 2018-06

**Authors:** Savandalath Phouangsouvanh, Mayfong Mayxay, Valy Keoluangkhot, Manivanh Vongsouvath, Viengmon Davong, David A.B. Dance

**Affiliations:** a Infectious Diseases Ward, Mahosot Hospital, Vientiane, Lao People’s Democratic Republic; b Faculty of Postgraduate Studies, University of Health Sciences, Vientiane, Lao People’s Democratic Republic; c Lao–Oxford–Mahosot Hospital–Wellcome Trust Research Unit (LOMWRU), Microbiology Laboratory, Mahosot Hospital, Vientiane, Lao People’s Democratic Republic; d Centre for Tropical Medicine & Global Health, Nuffield Department of Clinical Medicine, University of Oxford, Oxford, UK; e Faculty of Infectious and Tropical Diseases, London School of Hygiene and Tropical Medicine, London, UK

**Keywords:** *Neisseria gonorrhoeae*, Gonorrhoea, Antimicrobial susceptibility, Antimicrobial resistance, Laos, Lao PDR

## Abstract

**Objectives:**

The aim of this study was to determine the antimicrobial susceptibility of *Neisseria gonorrhoeae* isolates in the Lao People’s Democratic Republic (Laos).

**Methods:**

A total of 165 gonococcal isolates (1.3%) were obtained from 12 281 genital samples routinely submitted to a diagnostic laboratory in Vientiane, Laos, between 2011 and 2015. Susceptibility to five antibiotics was determined by the standard disk diffusion method for 158 of the isolates.

**Results:**

Rates of resistance to penicillin (by β-lactamase production), tetracycline and ciprofloxacin were 89.9%, 99.4% and 84.8%, respectively. All isolates were susceptible to ceftriaxone and spectinomycin.

**Conclusions:**

The situation in Laos is similar to that in neighbouring countries; this fortunately means that the latest Lao national guidelines for treating gonorrhoea should still be effective.

## Highlights

• There have been few studies on the susceptibility of *Neisseria gonorrhoeae* from Laos.
• 158 *N. gonorrhoeae* isolates from Vientiane were tested by the disk diffusion method.
• Rates of resistance to penicillin (by β-lactamase production), tetracycline and ciprofloxacin were 89.9%, 99.4% and 84.8%, respectively.
• All isolates remained susceptible to ceftriaxone and spectinomycin.
• Ceftriaxone is recommended as first-line treatment for gonorrhoea in the most recent Lao national guidelines.

## 1 Introduction

There are limited data on the antimicrobial susceptibility of *Neisseria gonorrhoeae* isolates in the Lao People’s Democratic Republic (Laos). This is largely because there are very few diagnostic microbiology laboratories in the country. Sexually transmitted infections (STIs) are thus usually managed empirically based on national guidelines [1]. Previous reports based on relatively small numbers of isolates have suggested high rates of antibiotic resistance in Lao isolates of *N. gonorrhoeae* [2], [3], [4], [5], [21], comparable with resistance rates in the neighbouring countries of Thailand [6], Vietnam [7], Myanmar [8] and southern China [9].

Routine culture for *N. gonorrhoeae* on all genital tract samples received in the microbiology laboratory of Mahosot Hospital (Vientiane, Laos) was introduced in December 2011; previously, diagnosis of gonorrhoea had relied on microscopy alone. Here we report the results of culture, including antimicrobial susceptibility testing, over a 5-year period (2011–2015).

## 2 Materials and methods

### 2.1 Patients and sample collection

Female patients with vaginal discharge who visited the gynaecology outpatient department (OPD) of Mahosot Hospital were examined by the ward gynaecologists or residents, and vaginal or endocervical swabs were taken and sent immediately (within 2 h) to the microbiology laboratory in Amies transport medium with charcoal (Oxoid Ltd., Basingstoke, UK). Male patients with urethral discharge who visited the general OPD of the hospital were referred to the microbiology laboratory for sample collection by laboratory staff.

### 2.2 Culture for *Neisseria gonorrhoeae*

Swabs were streaked on the surface of selective culture medium for *N. gonorrhoeae* [Oxoid GC agar base (CM0367) + 1% haemoglobin + Vitox + VCAT antibiotic supplement; Oxoid Ltd.] and were incubated at 35–37 °C in air enriched with 5% CO_2_. Plates were examined after 18–24 h and were re-incubated for a further 24 h if they were culture-negative. *Neisseria gonorrhoeae* was suspected if the plate grew oxidase-positive Gram-negative diplococci and was confirmed by biochemical (API NH Kit; bioMérieux, Basingstoke, UK) and serological (Remel™ Phadebact™ Monoclonal GC Test; Launch Diagnostics Ltd., Longfield, UK) methods.

### 2.3 Antimicrobial susceptibility testing

Isolates confirmed as *N. gonorrhoeae* were tested for antimicrobial susceptibility by the disk diffusion method on the medium described above without the selective supplement and incubation at 35–37 °C in 5% CO_2_ for 20–24 h. Results were interpreted according to the Clinical and Laboratory Standards Institute (CLSI) disk diffusion standards current at the time [10]. *Neisseria gonorrhoeae* ATCC 49226 was used as a control, which was tested weekly. The tested antibiotics were ceftriaxone (30 μg), spectinomycin (100 μg), ciprofloxacin (5 μg), penicillin G (10 U) and tetracycline (30 μg). β-Lactamase detection was undertaken using Cefinase disks (BD BBL™; Zuellig Pharma, Bangkok, Thailand).

### 2.4 Analysis

Data were entered into Microsoft Excel (Microsoft Corp., Redmond, WA) and analyses were done using STATA v.12.0 (StataCorp LP, College Station, TX).

## 3 Results

### 3.1 *Neisseria gonorrhoeae* culture

A total of 12 281 genital samples were received between 2011–2015, of which 12 259 (99.8%) were vaginal or endocervical swabs and only 22 (0.2%) were male urethral swabs. Patient age ranged from 2–83 years (median 30 years). Of the patients, 81.0% were from Vientiane Capital, 13.1% were from other provinces and no information about residence was available for 5.9%. Overall, *N. gonorrhoeae* was cultured from 165 (1.3%) of the 12 281 samples, including 9 (40.9%) of 22 urethral swabs and 156 (1.3%) of 12 259 vaginal swabs. Gram-negative diplococci were seen on microscopy in 46 samples (27.9% of culture-positive samples), of which all but one grew *N. gonorrhoeae* (Table 1).

**Table 1:** Detection rates by microscopy and culture, according to patient sex.

Microscopy	*Neisseria gonorrhoeae* culture				Total
	Growth		No growth		
	Female	Male	Female	Male	
Gram-negative diplococci not observed	119	1	12 102	13	12 235
Gram-negative diplococci observed	37	8	1	0	46

### 3.2 Antimicrobial susceptibility testing

Disk diffusion susceptibility results were available for 158 of the 165 isolates. All of the isolates were susceptible to ceftriaxone and spectinomycin. For ciprofloxacin, 134 isolates (84.8%) were resistant, 21 (13.3%) were classified as intermediate and only 3 (1.9%) were susceptible (Table 2). All isolates were classified as either intermediate or resistant to penicillin and tetracycline and were positive for β-lactamase production.

**Table 2:** Antimicrobial susceptibility patterns of *Neisseria gonorrhoeae.*

Antimicrobial agent	No. (%) of isolates		
	Susceptible	Intermediate	Resistant
Ceftriaxone	158 (100)	0 (0)	0 (0)
Spectinomycin	158 (100)	0 (0)	0 (0)
Ciprofloxacin	3 (1.9)	21 (13.3)	134 (84.8)
Penicillin	0 (0)	16 (10.1)	142 (89.9)
Tetracycline	0 (0)	1 (0.6)	157 (99.4)

## 4 Discussion

This is the first study in Laos to look at the value of introducing routine culture for *N. gonorrhoeae* of genital samples submitted to a diagnostic laboratory. The overall yield from samples from females was relatively low (1.3%), but urethral swabs from men with urethral discharge, although few in number, were frequently positive (9/22; 40.9%). Genitourinary medicine specialists do not exist in Laos and therefore samples were submitted by clinicians without specific training in specimen collection techniques, and the yield might have been increased had all women had urethral and endocervical swabs taken as opposed to ‘high vaginal’ swabs [11].

In this population, the overall prevalence of gonorrhoea (1.3%) was lower than that in a previous study in Laos (3.7%) [5]. This latter study specifically recruited women aged between 15–49 years attending the gynaecology OPD for the first time, a group that might be expected to have a higher prevalence of gonococcal infection than the unselected genital samples submitted to the laboratory in the current study [12]. None the less, introduction of culture into routine laboratory testing of genital samples at Mahosot Hospital enabled the confirmation of gonorrhoea in 120 patients in whom it would not have been detected by microscopy alone. It is well known that microscopy has a sensitivity of 95% in males but only 40–60% in females compared with 85–100% for culture [13], [14]. This is particularly important in women in whom gonococcal infection is frequently asymptomatic [5] but 10–20% are at risk of complications such as pelvic inflammatory disease and infertility if infection goes undiagnosed and therefore untreated [11], [15]. Molecular diagnostics, which have even greater sensitivity for the detection of *N. gonorrhoeae*, are not yet widely available in Laos.

As expected, resistance to penicillin and tetracycline was very common (89.9% and 99.4%, respectively). These drugs have not been recommended for the treatment of gonococcal infection for many years because of the known high resistance rates amongst gonococci worldwide [11].

This study also found that 84.8% of Lao *N. gonorrhoeae* were resistant to ciprofloxacin, with only 1.9% fully susceptible. This is a higher proportion than found in previous studies of smaller numbers of isolates from Laos: reduced quinolone susceptibility rates were reported to be 20% in 2000–2001 [5], 75.9% in 2002 [2], 65.5% in 2005 [3], 33% in 2007 [4] and 11% in 2008 [4]. In another study in Laos, ciprofloxacin resistance in *N. gonorrhoeae* was found to increase from 11.9% in 2000 to 69.5% in 2002 [21]. We suggest that *N. gonorrhoeae* from Laos should be considered likely to be quinolone-resistant unless proven otherwise. This is similar to the situation in Thailand [6], Vietnam [7], Myanmar [8], China [9], Bangladesh [16] and many other regions [17], [18], [19], [20]. Therefore, ciprofloxacin should no longer be used for treatment of gonococcal infection. Unfortunately, the current national guidelines for Laos, issued in 2012, include ciprofloxacin, ofloxacin and norfloxacin as possible first-line treatments for gonorrhoea [1]. These agents are no longer recommended in updated national guidelines produced in 2016, with only ceftriaxone, cefixime or spectinomycin recommended as first-line agents. These recommendations are consistent with our findings. Although single isolates of *N. gonorrhoeae* were reported to be resistant to ceftriaxone and spectinomycin in the study of Phanthouamath et al. [21] these results were not independently confirmed and there is no evidence from our study that such strains have persisted in Vientiane. Unfortunately, these updated guidelines have not yet been issued and distributed to clinicians.

There were a number of limitations of this study, particularly the fact that it was performed at only one site, in the Lao capital Vientiane, and so it is unknown whether significant regional variations in gonococcal susceptibility occur throughout Laos. The extremely small number of male patients in this study reflects the fact that when they have STI symptoms, they usually visit the general OPD or the dermatology centre where urethral samples are not usually taken for laboratory investigation. In addition, because of the retrospective laboratory-based nature of the study, it was not possible to collect data regarding risk factors or other epidemiological features that may have been associated with specific patterns of resistance, nor did we attempt to undertake typing to investigate chains of transmission. None the less, the uniformly high rates of resistance to penicillin, tetracyclines and quinolones make it relatively unlikely that such associations would have been found.

In conclusion, the majority of *N. gonorrhoeae* in Laos are resistant to ciprofloxacin, penicillin and tetracycline but remain susceptible to ceftriaxone and spectinomycin. Updated national guidelines for the management of STIs, including gonorrhoea, should be issued and implemented as soon as possible, and continued surveillance is essential to detect the emergence of further resistance.
